# Ventral and dorsal aspects of the inferior frontal-occipital fasciculus support verbal semantic access and visually-guided behavioural control

**DOI:** 10.1007/s00429-023-02729-5

**Published:** 2023-12-09

**Authors:** Tirso R. J. Gonzalez Alam, Juan Cruz Arias, Elizabeth Jefferies, Jonathan Smallwood, Alexander Leemans, Julian Marino Davolos

**Affiliations:** 1 Department of Psychology and York Neuroimaging Centre, University of York, York, UK; 2 Universidad Nacional de Córdoba, Córdoba, Argentina; 3 Department of Psychology, Queen’s University, Kingston, ON Canada; 4 Image Sciences Institute, University Medical Center Utrecht, Utrecht, The Netherlands; 5 School of Psychology, Bangor University, Bangor, UK

**Keywords:** Inferior frontal-occipital fasciculus, DTI, Tractography, Semantic access, Inhibition

## Abstract

The Inferior Frontal Occipital Fasciculus (IFOF) is a major anterior-to-posterior white matter pathway in the ventral human brain that connects parietal, temporal and occipital regions to frontal cortex. It has been implicated in a range of functions, including language, semantics, inhibition and the control of action. The recent research shows that the IFOF can be sub-divided into a ventral and dorsal branch, but the functional relevance of this distinction, as well as any potential hemispheric differences, are poorly understood. Using DTI tractography, we investigated the involvement of dorsal and ventral subdivisions of the IFOF in the left and right hemisphere in a response inhibition task (Go/No-Go), where the decision to respond or to withhold a prepotent response was made on the basis of semantic or non-semantic aspects of visual inputs. The task also varied the presentation modality (whether concepts were presented as written words or images). The results showed that the integrity of both dorsal and ventral IFOF in the left hemisphere were associated with participants’ inhibition performance when the signal to stop was meaningful and presented in the verbal modality. This effect was absent in the right hemisphere. The integrity of dorsal IFOF was also associated with participants’ inhibition efficiency in difficult perceptually guided decisions. This pattern of results indicates that left dorsal IFOF is implicated in the domain-general control of visually-guided behaviour, while the left ventral branch might interface with the semantic system to support the control of action when the inhibitory signal is based on meaning.

**Supplementary Information:**

The online version contains supplementary material available at 10.1007/s00429-023-02729-5.

## Introduction

The Inferior Frontal Occipital Fasciculus (IFOF) is a major anterior-to-posterior white matter pathway in the ventral human brain. It connects occipital, parietal and posterior temporal regions, implicated in word and object recognition, to prefrontal areas associated with cognitive control, semantic retrieval and speech production (Caverzasi et al. 2014; Duffau et al. 2013; Martino et al. 2010). In line with this functional anatomy, IFOF has long been associated with language and semantic cognition (Almairac et al. 2015; Binder et al. 2009; Han et al. 2013; Sierpowska et al. 2019). Stimulation of IFOF during neurosurgery elicits semantic errors in picture naming tasks (Duffau et al. 2005, 2008), while damage to IFOF is associated with semantic impairment (Han et al. 2013; Souter et al. 2022; Surbeck et al. 2020). Similarly, variation in the integrity of IFOF in healthy participants is associated with performance on lexical-orthographic tasks (Vandermosten et al. 2012) and bilingual aspects of language (Mohades et al. 2012). Nevertheless, there are many unresolved questions about the contribution of IFOF to cognition, including whether this tract supports both conceptual and visually-guided (non-conceptual) aspects of cognition, whether there are differences between semantic tasks employing pictures and words, and whether there are functional distinctions across subdivisions of IFOF.

Contemporary theoretical accounts emphasize that semantic cognition and language emerge from interacting component processes, which have dissociable neurocognitive bases (Lambon Ralph et al. 2017) and which might draw on different white matter tracts (Duffau et al. 2009, 2013; Moritz-Gasser et al. 2013; Rauschecker 2012). For example, a dorsal language pathway is thought to support acoustic − motor mappings and articulatory sequences, while a ventral language pathway supports semantic processing (Saur et al. 2008): by this account, conceptual retrieval draws on the interaction of heteromodal conceptual representations in an anterior temporal ‘hub’ with unimodal ‘spoke’ representations in visual and auditory cortex (Patterson et al. 2007). The inferior longitudinal fasciculus (ILF) links occipital cortex to anterior temporal regions and is consequently well-placed to support rapid and automatic access to heteromodal conceptual knowledge from visual inputs (Duffau et al. 2013; Herbet et al. 2018; Saur et al. 2008; Turken and Dronkers 2011). However, this pathway is thought to be insufficient for semantic cognition since we need to retrieve conceptual information in a flexible fashion to generate adaptive thoughts and behaviour and this is thought to involve the interaction of semantic knowledge with control processes. Recent studies have suggested that a distributed network including posterior middle temporal gyrus and anterior inferior frontal cortex is important for our capacity to focus on semantic information that is relevant to the evolving context or our current goals (Davey et al. 2016; Gao et al. 2021; Jackson 2021). This ‘semantic control network’ shows the strongest recruitment in fMRI studies when non-dominant aspects of knowledge are needed, or when there is ambiguity or conflict between concepts (Badre et al. 2005; Jackson 2021; Noonan et al. 2013; Thompson-Schill et al. 1997). Because IFOF connects posterior temporal to prefrontal regions, it is likely to play a critical role in the controlled application of knowledge during semantic cognition (Duffau et al. 2013; Giampiccolo and Duffau 2022; Nugiel et al. 2016). Patients with poor control of semantic cognition in the context of stroke aphasia have highly consistent damage to IFOF and ILF despite highly variable lesions affecting left inferior prefrontal or posterior temporal areas (Souter et al. 2022). Moreover, for phonological and semantic verbal fluency tasks with high executive demands, IFOF integrity is correlated with performance (Almairac et al. 2015; Nugiel et al. 2016).

IFOF also shows broader connectivity beyond semantic control regions, connecting occipital and parietal regions to broad swathes of lateral, anterior and ventral frontal regions (Catani and Thiebaut de Schotten 2008; Duffau et al. 2013) linked to cognitive control, decision making and speech production. Consequently, IFOF is likely to be important for controlled visually-guided cognition *beyond* the semantic domain; for example, Walsh et al. (2011) found that reduced microstructural integrity of the IFOF is associated with poor object working memory performance, and other studies have established a link between IFOF dysfunction and unilateral neglect (Herbet et al. 2017b; Karnath et al. 2011; Urbanski et al. 2008, 2011). Given this connectivity with visual cortex, it is also important to establish if the functional role of IFOF varies across semantic tasks involving pictures and words. If IFOF is critical for linking visual input regions to prefrontal cortex, this tract might play a greater role in semantic tasks that utilize picture inputs, such as picture naming and picture association. Alternatively, IFOF may support connections critical to the putative heteromodal semantic control network, linking posterior middle temporal gyrus to anterior portions of inferior frontal cortex: both of these regions respond to control demands across word and picture semantic tasks (Krieger-Redwood et al. 2015).

Research has also revealed dorsal and ventral subdivisions within IFOF, which may have different functional associations (Martino et al. 2010; Rollans and Cummine 2018; Roux et al. 2021; Sarubbo et al. 2013). Martino et al. (2010) found the superficial dorsal subcomponent of IFOF connects superior occipital regions associated with visually-guided action, plus posterior superior temporal and parietal regions, to posterior parts of inferior frontal gyrus: this pathway is potentially suited to the control of action and visual − spatial processing. In contrast, a deep ventral pathway was found to connect inferior occipital and ventral temporal regions implicated in object recognition, plus heteromodal posterior middle temporal gyrus, to diverse frontal lobe regions, including anterior and ventral regions within the default mode network and dorsolateral prefrontal regions associated with cognitive control: this pathway is therefore suited to supporting visually guided decision making and heteromodal semantic control. Sarubbo et al. (2013) corroborated this division of the IFOF into dorsal and ventral components, using a combination of the Klingler method and single-subject DTI, showing that the dorsal aspect terminates in the posterior inferior frontal gyrus, while the ventral component can be further divided in three minor bundles terminating in middle frontal gyrus and dorsolateral prefrontal cortex, lateral orbitofrontal cortex, and frontal pole. In line with this multi-bundle—as opposed to dichotomous—IFOF architecture, other studies have found a five-layer organization when streamline tractographies are initiated from frontal areas (e.g. Wu et al. 2016). Almairac et al. (2015) proposed that the ventral pathway might be important for semantic access, while the dorsal pathway supports phonology; the dorsal route is also implicated in reading and writing (Motomura et al. 2014). However, there are alternative functional interpretations. Rollans and Cummine (2018) found that Fractional Anisotropy (FA) in dorsal and ventral IFOF was linked to Go/No-Go and naming tasks respectively, suggesting the dorsal pathway may be crucial for controlled action driven by visual input beyond the domain of language or semantic cognition, while the ventral pathway supports more abstract conceptual behaviour.

Finally, given that language and semantic control are thought to be highly left-lateralised (Frost et al. 1999; Gonzalez Alam et al. 2019; Josse and Tzourio-Mazoyer Nathalie 2004), while visually-guided non-semantic cognition and domain-general executive processes are more bilateral (Bartolomeo and Seidel Malkinson 2019; Duncan 2010; Hellige and Michimata 1989; Hugdahl et al. 2015), there may be important functional dissociations between left and right IFOF. Using tract-based spatial statistics (TBSS), Rollans et al. (2017) found that IFOF was associated with gross picture naming differences in the left hemisphere, but more subtle differences in naming performance in the right hemisphere. Herbet et al. (2017a, b) showed that direct electrical stimulation of the IFOF in the right hemisphere is associated with poorer performance in non-verbal semantic processing. Moreover, in a meta-analysis performed by Vigneau et al. (2011), the cognitive load of semantic tasks determined the contribution of right IFOF: when the task involved working memory to manipulate verbal content, or the capacity to switch between categories in a verbal fluency test, the association with right IFOF was stronger.

In the current study, we investigated these diverse hypotheses about the functional relevance of subdivisions of left and right IFOF using a semantic Go/No-Go task. In semantic conditions, decisions to produce or withhold a prepotent button press response were made on the basis of conceptual content (whether a visually-presented stimulus was an animal or a manmade object), while in non-semantic conditions, these decisions were based on the shape of the box enclosing scrambled images, such that semantic access was not necessary for the task. The semantic conditions also compared written words and pictures, to allow us to consider the extent to which effects related to language, transmodal aspects of semantic cognition or visual semantic processes necessary only for pictorial tasks. Semantic efficiency should show an association with tracts that link visual inputs to conceptual regions needed to select an appropriate response based on meaning, particularly for verbal trials, since written word inputs do not contain any superficial cues to the behaviourally relevant categorical distinction, unlike pictures. In contrast, the inhibition efficiency for both semantic and non-semantic Go/No-Go tasks is expected to relate to tracts that connect visual regions to dorsal parts of prefrontal cortex supporting action selection and inhibition. In summary, given the distinct anatomy of dorsal and ventral IFOF, we expected dorsal IFOF to be relevant to visually-guided Go/No-Go behaviour across semantic and non-semantic domains, while ventral IFOF was expected to be associated with controlled semantic behaviour.

## Methods

This study constitutes a re-analysis of behavioural data originally published in Gonzalez Alam et al. (2018), which focussed on individual differences in intrinsic functional as opposed to structural connectivity. The study was approved by the University of York Neuroimaging Centre and by the Department of Psychology ethics committees. We reproduce the relevant sections from the original paper below.

### Participants

The sample consisted of 60 right-handed, native English-speakers with normal or corrected to normal vision, with no history of neurological or psychiatric illness (mean age = 20.2, SD = 2.1, range = 18–27 years, 37 females). All participants were volunteers recruited from the University of York and provided informed consent. This sample was collected as part of a larger project investigating links between individual differences in neuroanatomy, neural function, and cognition. Participants also completed a large battery of memory, executive and perceptual tests, as well as resting-state fMRI (Evans et al. 2020; Gonzalez Alam et al., 2021, 2022; Karapanagiotidis et al. 2017; Sormaz et al. 2018; Turnbull et al. 2018; Vatansever et al. 2017; Wang et al. 2018), but this data falls outside the scope of the present study.

### Materials

#### Go/No-Go paradigm

The participants took part in a Go/No-Go task designed to probe semantic inhibition. Each trial consisted of a fixation cross, followed by the stimulus. The duration of the fixations and stimuli were jittered between 0.5-1s and 0.75–1.25s for fixation and stimulus, respectively. The stimuli consisted of pictures/words framed by a box that was slanted to different degrees (slight slant, medium slant or pronounced slant).

**Fig. 1:**
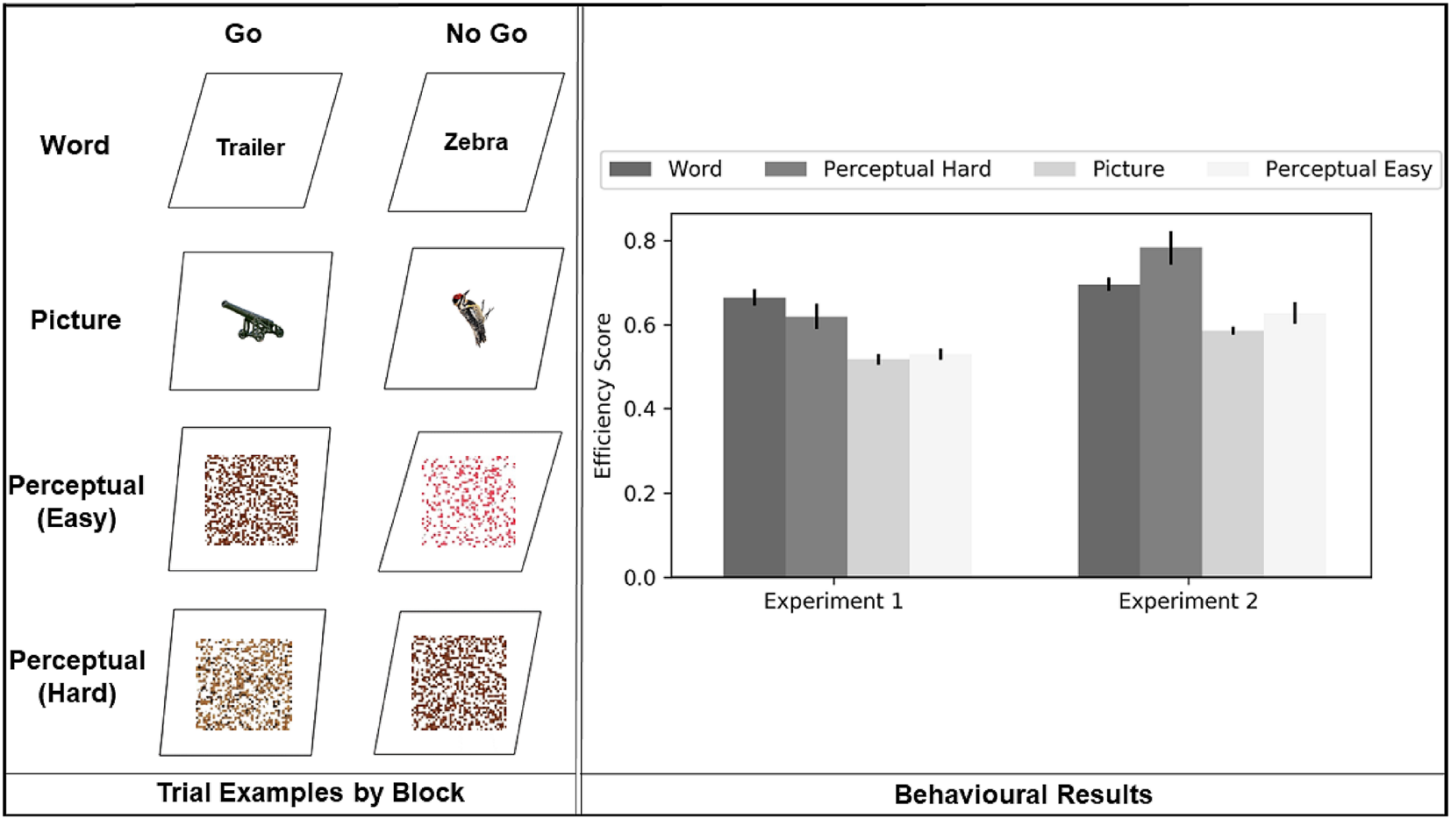
The left-hand panel depicts example stimuli per block. In WORD blocks, participants pressed for words denoting manmade objects and withheld this response for words denoting animals. In PICTURE blocks, participants pressed for pictures of manmade objects and withheld this response for pictures of animals. In Perceptual blocks, participants pressed for slightly slanted boxes and withheld this response for more strongly slanting boxes. Difficulty in the Perceptual trials was manipulated by adjusting the size of the slant. The right-hand panel shows behavioural results for the Go/No-Go paradigm expressed as inverse efficiency scores (a proportion of reaction time divided by accuracy); higher scores indicate greater difficulty. The error bars depict the standard error of the mean. In Experiment 1 (previously reported in Gonzalez Alam et al. 2018), the task was performed inside the scanner, while in Experiment 2 (reported here together with DTI analysis), it was performed outside

The task was divided into three blocks: in the ‘Word’ blocks, the participants saw a series of words referring to either animals or manmade objects, while in the ‘Picture’ blocks, they saw pictures depicting these same categories; their task was to press a button every time they saw a word or picture referring to a manmade object (Go event), and refrain from pressing when they saw an animal (No-Go event). In the ‘Perceptual’ (non-semantic) blocks, stimuli were scrambled images generated from the word and picture stimuli ensuring that basic features like luminance were constant across the experiment (See the ‘Stimuli Generation’ section for details). In this condition, participants were asked to inhibit responses when they saw that the framing box was more slanted than usual (No-Go event) and to press the button for the usual, slight degree of slant (Go event). This last condition was further subdivided in Easy and Hard trials based on the degree of slant: The Easy trials involved discriminating between slight and pronounced slants, while the Hard trials involved discriminating between the slight and medium slants (this was harder to do, as there was only a slight difference between them). This manipulation was included to provide perceptual decisions that matched in difficulty to both word and picture semantic trials. Examples of the Go and No-Go trials, as well as the behavioural results from the paradigm are presented in Fig. 1.

The task was organised in six blocks (two for each condition) containing 46–54 stimuli each, with the order of blocks counterbalanced across participants. Each block contained 80% Go events and 20% No-Go events. We divided these into two 3-block runs, each lasting 13 min, with an optional short break between the runs. The distribution of Go and No-Go events within the blocks was pseudorandomised, with 1–6 Go events between No-Go events. Each block started with a cue to inform the participant which type of stimuli to expect, and ended with a screen informing the participant they had a 5 s break before the next block. Our design made it necessary to trade off the number of No-Go events with the strength of the inhibition effect (which is maximised by having predominately Go events and relatively few No-Go events). We opted for approximately 20 No-Go events against 80 Go events per condition.

#### Stimuli generation

In order to ensure the stimuli could be clearly distinguished as manmade or animal, we presented images from the *Bank of Standardized Stimuli* (Brodeur et al. 2010, 2014) to four native British English speakers, who provided as many names as possible for each picture, and decided if the item belonged to the category of animal or manmade object. Based on this, we chose a subset of pictures given a single non-ambiguous word as a name (i.e., with a single meaning). This provided 174 pictures of manmade objects and 51 of animals. Subsequently, we used independent samples t tests to verify that the names assigned to the manmade objects and animals did not differ significantly in lexical frequency and letter length using Celex implemented in N-Watch (Davis 2005). There were no significant differences in lexical frequency (manmade objects: M = 13.1 counts per million, SD = 22.6; animals: M = 12.0, SD = 27.5; t(219) < 1), or letter length (manmade objects: M = 6.2, SD = 2.1; animals: M = 6.1, SD = 2.2; t(219) < 1). The scrambled images were derived from these selected picture and word trials. We submitted the original pictures to a scrambler that broke them down in 160 equilateral ‘tiles’, and then randomly assigned a place to each tile to create a scrambled image of 40 × 40 tiles where no meaning was discernible. We did the same for the visually-presented words used in the word condition. The resulting scrambled pictures constituted the stimuli of the Perceptual trials.

### Image acquisition

Neuroimaging data were acquired using a 3T GE HDx Excite MRI scanner utilising an eight-channel phased array head coil (GE) tuned to 127.4 MHz, at the York Neuroimaging Centre, University of York. Structural MRI acquisition in all participants was based on a T1-weighted 3D fast spoiled gradient echo sequence (TR = 7.8 ms, TE = minimum full, flip angle 20°, matrix size = 256 × 256, 176 slices, voxel size = 1.13 × 1.13 × 1 mm). An intermediary FLAIR scan with the same orientation as the functional scans was collected to improve the co-registration between subject-specific structural and functional scans. The diffusion MRI scan was 13 min in duration. A single-shot pulsed gradient spin-echo echo-planar imaging sequence was used with the following parameters: *b* = 1000 s/mm^2^, 45 directions, 7 T2-weighted EPI baseline scans (b0), 59 slices, FOV = 192 × 192 mm^2^, TR = 15 s, TE = 86 ms (minimum full), voxel size = 2 × 2 × 2 mm^3^, matrix = 96 × 96. The structural data used in this study has previously been utilised by (Karapanagiotidis et al. 2017). Full details of this sample, as well as all the parameters of the diffusion-weighted imaging sequence used, are provided here: http/fcon_1000.projects.nitrc.org/indi/pro/eNKI_RS_TRT/FrontPage.html.

### Image analysis

#### Structural connectivity analysis

The MR images were processed using MATLAB R2014a and ExploreDTI 4.8.6 (Leemans et al. 2009). First, Gibbs ringing artifacts in the b0 images were corrected with the total variation method (Perrone et al. 2015). Then, subject motion and eddy current induced artefacts were corrected by applying an affine registration of the DWI to the b0 image. The b-matrix was accordingly rotated (Leemans and Jones 2009). The DWI were non-rigidly registered to the T1 image to correct for distortions due to echo-planar imaging. Finally, the Fibre Assignment by Continuous Tracking (FACT) algorithm (Mori et al. 1999) was used to perform whole-brain DTI-based deterministic tractography, with the following parameters: fractional anisotropy threshold for streamline initiation and continuation = 0.2, length threshold 10–500 mm, step size = [2 2 2] mm, angle threshold = 35°.

**Fig. 2:**
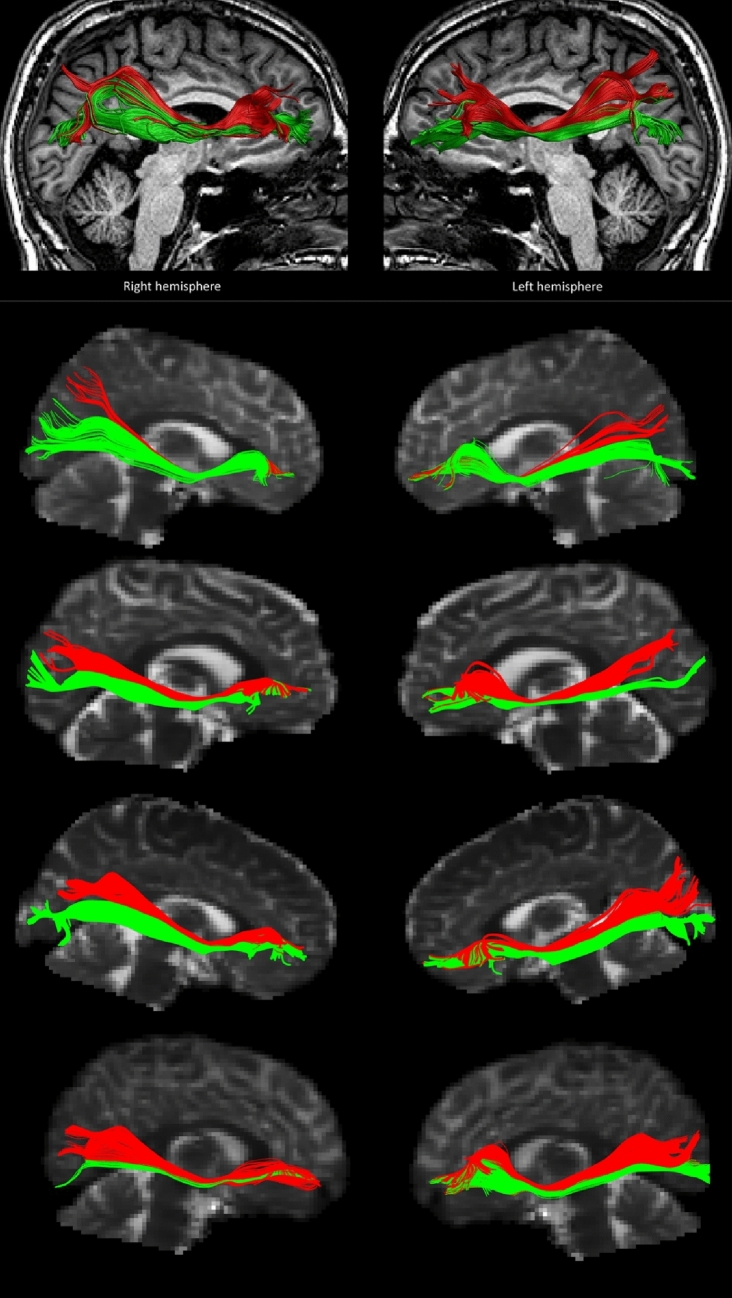
The top panel illustrates the dorsal and ventral tracts of the inferior fronto-occipital fasciculus resulting from the tractography for one of our cases, overlaid on the structural image of the case. The bottom panel depicts four more examples of the dorsal and ventral IFOF from our cases, overlaid on their non-diffusion weighted image to illustrate the inter-individual variability in these tracts. The dorsal IFOF is depicted in red, and the ventral IFOF in green

A manual tractography dissection method was applied, performed by one of the authors (JMD) with expertise in neuroanatomy. The methodology for in vivo tractographical dissection by region of interest drawing is thoroughly described in Wakana et al. (2004) and for tract labelling we followed the white matter atlas by Catani and Thiebaut de Schotten (2008). Since we performed manual dissections by an expert, each ROI was drawn following the idiosyncrasies of the participant’s white matter tract anatomy. The advantage of this method over automated methods is that it does not introduce spurious tracts and allows us to address individual differences in anatomy (Bach et al. 2014). The general approach to perform the IFOF dissection and sub-division was as follows. As a first step, we utilised two AND ROIs placed in coronal slices to define the full IFOF tract: an occipital ROI placed near the posterior third of the occipital lobe, and a frontal ROI placed near the anterior termination of the corpus callosum, as well as a NOT ROI placed in a sagittal slice separating the hemispheres to exclude spurious cross-hemisphere fibres (see Figure S2). We utilised Boolean algebra to extract all fibres that satisfied our ROIs constraints and compared the resulting tract with the IFOF template from the white matter atlas by Catani and Thiebaut de Schotten (2008) to verify the quality of the dissection. Once the full IFOF tract had been extracted, we segmented it into a dorsal and ventral aspect by placing two distinct AND ROIs in coronal occipital slices (since these tracts show clear separation at this location) separating the ventral fibre bundle parallel to the optic radiation from the dorsal fibre bundle that runs parallel to the vertical occipital fasciculus (see Figure S3). These, in combination with the aforementioned sagittal NOT and frontal AND ROIs were used to extract the ventral and dorsal IFOF respectively as follows. The ventral occipital AND, frontal AND, and sagittal NOT ROIs were used to define the ventral IFOF (Figure S4), whilst the dorsal occipital AND, frontal AND, and sagittal NOT ROIs were used to define the dorsal IFOF (Figure S5). Depending on the case, further NOT ROIs were used to eliminate spurious fibres from adjacent tracts as needed. This procedure was performed separately for each hemisphere. We provide an illustration of this approach applied to an example case in the Supplementary Materials (see section “Approach used to segment the Inferior Frontal − Occipital Fasciculus into its dorsal and ventral components applied to an example case”). We then computed average FA for each tract. The use of tract-average microstructural statistics is widespread in the tractography literature (for recent examples, see: Boukadi et al. 2019; Debarle et al. 2017a; Ezzati et al. 2016; Mole et al. 2016). Since we did not include parietal ROIs, our reconstructed tracts show limited streamlines in this region. Examples of the dissection of the IFOF into its dorsal and ventral subcomponents can be consulted in Fig. 2.

#### Analysis overview for behaviour-tract associations

Using repeated-measures ANCOVA in SPSS, we first examined the relationship between task structure (i.e. differences between the four task conditions) and tract integrity (including left and right dorsal and ventral IFOF as covariates). Having established significant interactions with task condition for left but not right IFOF, we performed a subsequent ANCOVA examining the interaction of IFOF integrity in LH with task contrasts that probed effects of modality, difficulty and condition (semantic versus perceptual). Finally, we examined hemispheric differences in IFOF tract integrity by assessing interactions between hemisphere and task contrasts using the same approach. Significant results from these ANCOVAs were further characterised with post hoc Pearson’s product − moment coefficient correlations to establish which task conditions or contrasts were contributing to the effects.

## Results

### Behavioural results

Following Gonzalez Alam et al. (2018), we combined reaction time (RT) on Go events and the accuracy of participants’ responses on No Go events into an inverse efficiency score (a ratio of a participant’s RT divided by accuracy) for each condition to use as an index of inhibition efficiency. Before proceeding with any further analyses, we imputed outliers that were beyond z =  ± 1.96 from the mean with the cutoff point of 1.96. Table 1 provides descriptive statistics for RT and accuracy, while the inverse efficiency score is shown in Fig. 1 (see Methods section). The data from Experiment 2 were used in the analysis of structural connectivity, while the data in Experiment 1 is from the same task recorded during fMRI by Gonzalez-Alam et al. (2018).

**Table 1:** Response time and accuracy for the behavioural data

Condition	Experiment 1 (*n* = 27)		Experiment 2 (*n* = 60)	
	RT	Accuracy	RT	Accuracy
Word	0.43 (0.04)	66.27 (12.51)	0.51 (0.05)	75.65 (11.97)
Picture	0.42 (0.07)	81.33 (12.35)	0.51 (0.06)	87.77 (8.87)
Perceptual Easy	0.41 (0.05)	79.05 (9.06)	0.49 (0.07)	81.06 (16.19)
Perceptual Hard	0.42 (0.06)	71.12 (17.98)	0.50 (0.07)	70.22 (17.87)

Note. Means with standard deviations in parentheses. RT on Go trials (i.e., when a response was required) is shown in seconds. Accuracy on No-Go trials (i.e., the successful suppression of a pre-potent response) is given as a percentage of trials

We analysed inhibition efficiency indexed through inverse efficiency scores to examine difficulty effects, since difficult trials should have worse efficiency. In the data from Experiment 2, analysed together with structural connectivity in the present study, the Word trials were more demanding than the Picture trials (t(59) = -9.38, *p* < 0.0001, Cohen’s d = 1.21), and the Perceptual Hard trials were more demanding than the Perceptual Easy trials (t(59) = 5.09, *p* < 0.0001, Cohen’s d = 0.65). Behavioural performance was matched for Word and Perceptual Hard conditions (t(59) = − 2.53, *p* = 0.056, Cohen’s d = 0.32) and Picture and Perceptual Easy conditions (t(59) = − 1.74, *p* = 0.352, Cohen’s d = 0.22). All p values are Bonferroni-corrected for 4 multiple comparisons. These results are shown in Fig. 1 (bars labelled “Experiment 2”). This pattern was replicated for accuracy (see accuracy analysis in Supplementary Materials).

In Experiment 1 (recorded during task-based fMRI and not considered in detail here), the Word trials were more demanding than the Picture trials: t(26) = 8.83, *p* < 0.0001 (see Fig. 1). Behavioural performance was matched for Word and Perceptual Hard conditions (t(26) = 1.68, *p* = 0.11), and Picture and Perceptual Easy conditions (t(26) = − 0.84, *p* = 0.41).

### Relationship between IFOF subdivisions and inhibition efficiency

We performed an one-way repeated measures ANCOVA to examine the association between tract integrity and inverse efficiency scores on the Go/No-Go task, including task condition as a factor with four levels, corresponding to Word, Picture, Perceptual Hard and Perceptual Easy conditions. We entered separate values for left and right dorsal and ventral IFOF (four covariates). The results revealed no significant overall effect of task (F(1.67, 92.1) = 2.1, *p* = 0.14). Left ventral and dorsal IFOF integrity interacted with task condition (LH ventral IFOF: F(1.67, 92.1) = 3.74, *p* = 0.035, η_p_^2^ = 0.064; LH dorsal IFOF: F(1.67, 92.1) = 7.06, *p* = 0.003, η_p_^2^ = 0.114). No significant associations were observed for right hemisphere tracts (*p* > 0.05). All effects are reported with Greenhouse − Geisser correction since the data violated the assumption of sphericity. The exact p values of significant and non-significant covariates for this, and the other two ANCOVAs reported in this section, can be consulted in Supplementary Table S1.

**Fig. 3:**
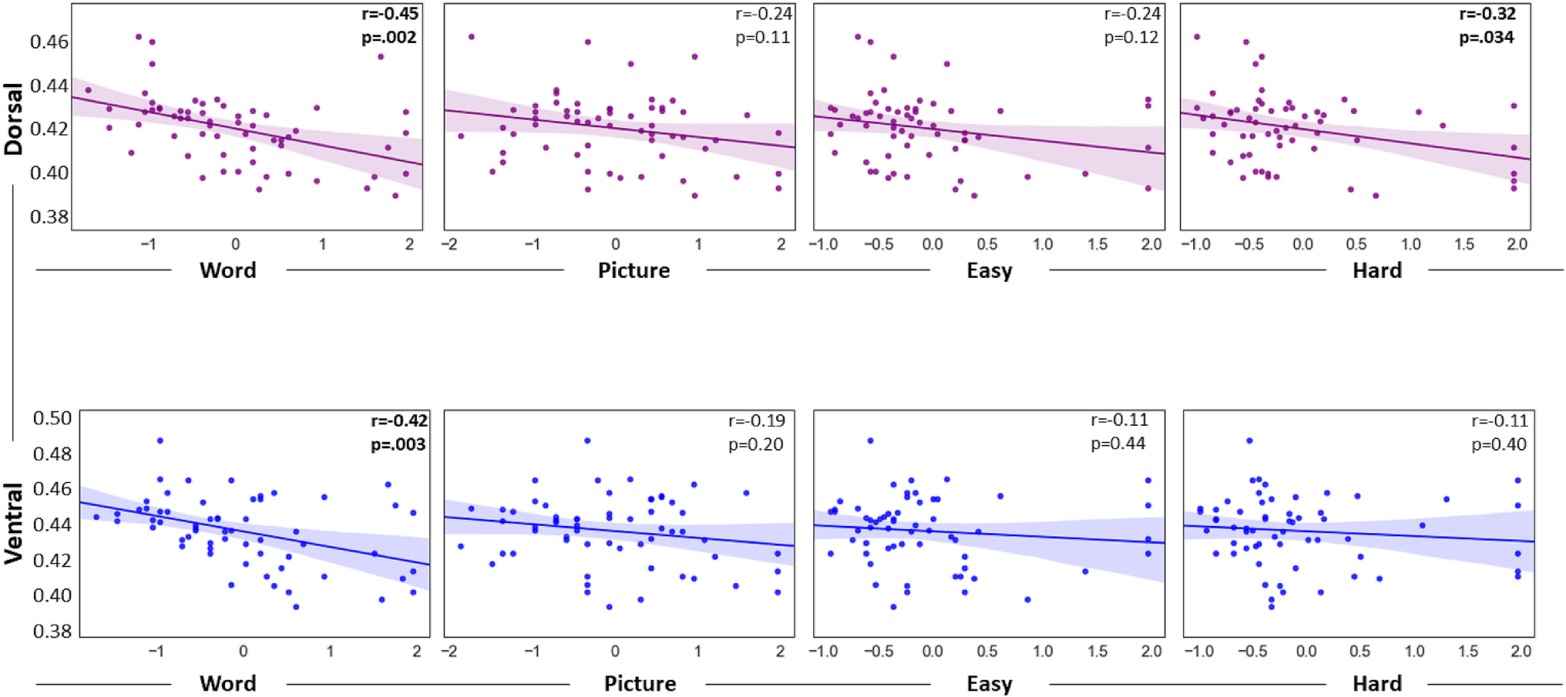
Scatterplots depicting the correlations between Dorsal and Ventral IFOF in LH (on the Y axis) and z-scored inverse efficiency scores for the Word, Picture, Perceptual Easy and Perceptual Hard conditions (on the X axis). The regression line and confidence interval are shown as illustration of the trend only

**Fig. 4:**
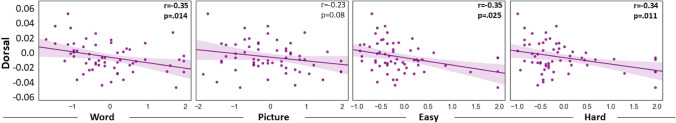
Scatterplots depicting the correlations between the LH—RH hemispheric difference in tract integrity for Dorsal IFOF (on the Y axis) and the z-scored inverse efficiency scores of the Word, Picture, Easy and Hard conditions (on the X axis)

Having observed significant interactions between inverse efficiency scores and both dorsal and ventral IFOF integrity in the LH, we asked which tasks show particularly strong associations with left dorsal and ventral tract integrity using post hoc Pearson’s product − moment correlations for each task separately. This revealed significant correlations for left dorsal IFOF with inverse efficiency scores in both Word (*r* = − 0.45, *p* = 0.002) and Perceptual Hard (*r* = − 0.32, *p* = 0.034) conditions. Left ventral IFOF showed a significant correlation with inverse efficiency scores in the Word condition only (*r* = − 0.42, *p* = 0.003). These results can be seen in Fig. 3. All correlation results reported here, and subsequently, are FDR-corrected to account for the number of task conditions, and all p values presented in all scatterplots in Figs. 3, 4 and S1 are Benjamini − Hochberg Adjusted p values; the exact p values and FDR correction can be consulted in Supplementary Table S2.

Given that we observed a significant association between hard perceptual trials and IFOF integrity for the dorsal but not ventral subdivision, we asked if this tract difference was significant. To assess this, we used the R package *cocor* (Diedenhofen and Musch 2015), which reports the results of 10 significance tests that compare the strength of two correlations. There was a significantly stronger correlation between performance on the hard perceptual task and dorsal IFOF integrity, compared with ventral IFOF integrity, regardless of the significance test used (all tests rejected the null hypothesis with p values between 0.017 and 0.021; the exact results of each test can be consulted in the Supplementary Materials).

Next, to test whether these interactions between task condition and the integrity of LH tracts were driven by effects of modality, cognitive domain or difficulty, we examined task contrasts. We calculated the difference between inverse efficiency scores for word and picture trials (word minus picture: modality contrast), for hard and easy perceptual trials (hard minus easy: difficulty contrast), and for semantic versus perceptual trials (word and picture minus hard and easy perceptual: semantic contrast). We performed an one-way repeated-measures ANCOVA with these differences between task conditions as a factor (three levels: modality, difficulty and semantic contrasts), and entered the same IFOF tract covariates as before (four covariates: left ventral, right ventral, left dorsal and right dorsal). There was no main effect of task contrast (F(1.37, 75.5) = 2.43, *p* = 0.112). There were significant yet distinct task contrast interactions for left ventral and dorsal IFOF (LH ventral IFOF: F(1.37, 75.5) = 4.6, *p* = 0.024, η_p_^2^ = 0.078; LH dorsal IFOF: F(1.37, 75.5) = 8.53, *p* = 0.002, η_p_^2^ = 0.134) but no interactions for RH tracts (*p* > 0.05). All effects are reported with a Greenhouse − Geisser correction. Post-hoc Pearson’s product − moment correlations, FDR-corrected, revealed a significant association between the effect of modality and the integrity of left dorsal and ventral IFOF (dorsal: *r* = − 0.4, *p* = 0.005; ventral: *r* = − 0.45, *p* = 0.002), as well as an association between the effect of difficulty and left dorsal IFOF (*r* = − 0.31, *p* = 0.03). The word task was harder than the picture task and consequently all individual differences associated with the integrity of left dorsal IFOF may reflect task difficulty; in contrast, the functional association with word decisions for left ventral IFOF cannot readily be explained by difficulty. These results can be seen in Figure S1 of the Supplementary Materials. All the non-significant correlations r and p values for these two analyses are provided in the figures.

Finally, we investigated potential hemispheric differences in the interaction between IFOF tract integrity and performance. This ANCOVA analysis included task condition as a factor (four levels: word, picture, hard, easy), and hemispheric differences in tract integrity for dorsal and ventral IFOF as covariates (LH–RH dorsal IFOF; LH–RH ventral IFOF). The main effect of task condition was significant (F(1.73, 98.4) = 18.5, *p* < 0.001, η_p_^2^ = 0.245). There was a significant interaction between hemispheric differences in the integrity of dorsal IFOF and task (F(1. 73, 98.4) = 6.09, *p* = 0.005, η_p_^2^ = 0.097). The interaction between hemispheric differences and task in the ventral IFOF approached significance (F(1. 73, 98.4) = 2.76, *p* = 0.076, η_p_^2^ = 0.046). All effects are reported with Greenhouse − Geisser correction. Post hoc Pearson’s product − moment correlations (FDR-corrected) revealed a significant correlation between hemispheric differences in dorsal IFOF and inverse efficiency scores in the word (*r* = − 0.35, *p* = 0.014), easy (*r* = − 0.35, *p* = 0.025) and hard conditions (*r* = − 0.34, *p* = 0.011). These results can be seen in Fig. 4. There was no significant correlation for the picture condition (*r* = 0.23, *p* = 0.08).

Analyses of hemispheric differences in tract strength for ventral and dorsal IFOF, and correlations between tracts and hemispheres, are provided in Supplementary Analyses (see section “Fractional Anisotropy Analysis”).

## Discussion

This study examined the associations between distinct IFOF subdivisions in the left and right hemispheres and behavioural inhibition guided by semantic versus perceptual properties of the stimuli. Inhibition efficiency showed a significant interaction with tract integrity for both the dorsal and ventral IFOF in the left hemisphere but there were no significant associations in the right hemisphere. In left dorsal IFOF, tract integrity interacted with the efficiency scores of the inhibition task across both semantic and non-semantic domains: there was an association with performance on both the Word and Hard Perceptual conditions; behaviourally, these were also the two hardest conditions. The integrity of the dorsal IFOF tract also predicted the magnitude of the effect of difficulty. Left ventral IFOF showed a somewhat different pattern: the integrity of this tract predicted performance on the Word task, with no association for non-semantic inhibitory control. This association with tract integrity was greater for the verbal than the picture-based semantic decisions—and this semantic modality effect could not be readily explained in terms of an influence of general task difficulty since left ventral IFOF did not show a difficulty effect for the non-semantic trials. Lastly, we investigated hemispheric differences in these tract-inhibition associations. All the task conditions, except Picture decisions, showed better performance in participants who had stronger left than right hemisphere IFOF tracts, although this pattern only reached statistical significance in the dorsal IFOF.

These findings are highly consistent with the proposal of functional differences between the dorsal and ventral subdivisions of the IFOF in the left hemisphere, with dorsal IFOF showing greater involvement in the regulation of difficult perceptual Go/No-Go trials, while ventral IFOF is engaged exclusively when inhibition is guided by abstract meaning (Rollans 2016). The semantic effect in the ventral IFOF was stronger for the verbal task, perhaps because decision-making in these trials required abstract categorical information to be more fully accessed. Unlike written words, the picture semantic trials provided visual feature cues about the category of each stimulus: for example, animals have shared visual features, such as eyes and tails, and the presence of these features even in the absence of conceptual identification could have been sufficient to drive an appropriate response to pictures. Tract integrity in dorsal IFOF, in contrast, was linked to the more difficult semantic and non-semantic decisions; like ventral IFOF, the dorsal subdivision showed stronger behavioural associations with word than picture performance, but there are alternative interpretations of this difference. First, the effect of modality in the semantic inhibition task in left dorsal IFOF may have reflected the greater need for orthographic to phonological conversion processes and/or visual − spatial processes needed to read words, since previous studies have previously implicated dorsal IFOF in reading (Motomura et al. 2014) and phonology (Almairac et al. 2015). However, this interpretation cannot explain the distinction between easy and harder non-semantic performance. An alternative interpretation is that left dorsal IFOF supports more difficult visually guided decision-making, as for both non-semantic decisions (are the lines of a box off vertical?) and semantic decisions (is the object an animal or manmade?), behavioural associations with left dorsal IFOF were stronger when the information needed for decision-making was less salient. Overall, left dorsal IFOF was associated with more demanding task conditions across domains (both word and hard perceptual trials), indicating that its function is not specific to language or semantic cognition; this pattern of results suggests that dorsal IFOF contributes to the ‘multiple demand network’ that supports domain-general executive processing (Duncan 2010, 2001). The functional distinction between dorsal and ventral IFOF is also consistent with the proposal that there are dissociable yet spatially proximal mechanisms supporting different aspects of cognitive control in the prefrontal cortex, including the possibility that the multiple demand network can be distinguished from semantic control mechanisms (e.g., Chiou et al. 2023; Gao et al. 2021; Whitney et al. 2011), and the observation that parcellations of resting-state connectivity patterns identify distinct networks linked to control (Dixon et al. 2018; Schaefer et al. 2018). The differences between dorsal and ventral IFOF reported here also fit well with the findings of Rollans (2016), who found a dissociation between visually guided inhibition and picture naming: however, the current study utilizes cleaner contrasts between similar task conditions to show this dissociation.

We also found evidence that our Go/No-go task was more reliant on the left hemisphere overall, for both semantic and non-semantic decisions. This is surprising since a functional neuroimaging study of the same task showed responses that were strongly right-lateralized in dorsolateral prefrontal cortex (Gonzalez Alam et al. 2018), alongside bilateral occipital − temporal activation. Only the contrast of semantic over non-semantic decisions revealed clusters in left prefrontal regions including in left inferior frontal gyrus; consequently, only semantic conditions of this task might be expected to be more reliant on white matter tracts projecting from occipital − temporal to prefrontal cortex in the left hemisphere. The unexpected hemispheric effects in the current study could reflect differences between tractography and BOLD fMRI, since left IFOF might be more important than right IFOF for some aspects of the task and yet connections between left and right prefrontal cortex could still result in a stronger response in the right hemisphere if, for example, right pre-supplementary motor and dorsolateral prefrontal regions play a stronger role in motor control (Aron et al. 2014; Cai et al. 2012; Hannah and Aron 2021; Rae et al. 2014). One relevant aspect of the task could be inhibition efficiency: whilst the right-lateralised activation observed in fMRI studies might be related to motor inhibition, our study used efficiency scores of an inhibition task as a metric. In support of this, Hirose et al. (2012) found a separation of inhibitory processes using an efficiency index: while the right hemisphere showed the usual neural substrates associated with response inhibition, inhibition efficiency specifically was associated with a set of structures in the left hemisphere, including temporal and frontal regions which might be subserved by the IFOF.

There are of course some limitations of our approach. First, the functional differentiation of the IFOF may extend beyond the left and right dorsal and ventral subdivisions examined here. In a study with diffusion data that resolved “kissing fibers” and with “high-angular resolution”, Wu et al. (2016) proposed five divisions of the IFOF, involving orbito-frontal, dorsolateral frontal, angular gyrus and marginal gyrus portions. Other researchers have proposed graded and continuous variation of the location of tract terminations across IFOF; by this view, while there are functional dissociations within IFOF in line with our data, this functional variation does not necessarily reflect distinct tract subdivisions (Weiller et al. 2021). Secondly, given that IFOF has been previously associated with the integrity of the semantic control network (Nugiel et al. 2016), which links heteromodal posterior middle temporal and inferior frontal regions implicated in the retrieval of non-dominant conceptual information, we cannot rule out the possibility of different tract integrity findings in studies that directly manipulate the need to control conceptual retrieval. Our semantic conditions required participants to use word and picture meaning to decide whether to press a button or withhold this response—but did not require conceptual retrieval itself to be controlled. Future studies could clarify whether distinct aspects of IFOF support (i) the control of meaning retrieval (using classic semantic control manipulations such as contrasts of weak and strong associations); (ii) the control of behaviour based on meaning (as in the semantic conditions of the current task) and/or (iii) the control of behaviour based on non-meaningful aspects of visual inputs (as in the Easy/Hard Perceptual conditions used here). This type of study might provide new information about the partial separation of the semantic control network from the domain-general multiple-demand network (Davey et al. 2016; Gao et al. 2021; Gonzalez Alam et al. 2018; Wang et al. 2020). Thirdly, the current investigation was restricted to examining subdivisions of the IFOF (since tract tracing for each individual participant is time-consuming and there are statistical limits on the number of tracts that can be investigated given our sample size). However, IFOF does not underpin semantic cognition alone and in future work, it will be important to consider how interactions between tract strengths for IFOF, uncinate fasciculus and ILF underpin distinct aspects of semantic and non-semantic cognition. A related issue is that the study did not include a control tract beyond the semantic and cognitive control domains. As a consequence, we cannot fully confirm the specificity of our findings. However, since semantic cognition and cognitive control both draw on highly distributed networks (e.g., Duncan 2010; Jackson 2021), it is challenging to confidently identify tracts that would not be expected to make *any* contribution to these functions. Finally, there are some potential methodological weaknesses in our analysis. We used an angle threshold of 35° to perform streamline tractography, in line with some earlier investigations that have used angles under 45° (Forkel et al. 2014; Wakana et al. 2007) and with strategies and recommendations given our tract of interest and resolution (Mori et al. 2002; Mori and Van Zijl 2002), yet other studies have used 60° (Caverzasi et al. 2014; Wu et al. 2016), or 45° (Hau et al. 2016). Using a smaller angle of threshold has some advantages (as well as potential disadvantages): the IFOF has a complex course that crosses other tracts, and a smaller angle is thought to make it easier to exclude fibres that are not part of IFOF (Thomas et al. 2014). Also, since IFOF is a relatively small tract, using a smaller angle threshold might allow for more detailed tract reconstruction (Mori and Van Zijl 2002). However, fibres targeting the superior parietal cortex and middle/superior frontal gyrus may be difficult to track with this parameter value, given their geometrical orientation.

In conclusion, using an individual differences approach in healthy participants, we show broad involvement of IFOF in both semantic cognition and visually guided decision-making. These findings are potentially consistent with recent neuroanatomical accounts which suggest that IFOF connects prefrontal cortex to both posterior heteromodal semantic regions in posterior temporal cortex (although the presence of these connections is variable across subjects) and visual regions in occipital and ventral temporal cortex (Catani and Thiebaut de Schotten 2008; Duffau et al. 2013; Giampiccolo and Duffau 2022; Martino et al. 2010; Nugiel et al. 2016).

### Supplementary Information

Below is the link to the electronic supplementary material.

Supplementary file1 (PDF 704 KB)

## Data Availability

Raw fMRI and behavioural data are restricted in accordance with ERC and EU regulations.

## Code Availability

The code used in the production of Figs. 3, 4 and S1, as well as Table S2 is available in the first author’s GitHub (https://github.com/TirsoRJGonzalez/GoNoGo_IFOF) and in an OSF collection, together with the detailed ANCOVA and Pearson correlation results (https://osf.io/gxah6/). The code has been modified from the production version to remove all information that could potentially identify participants.
