# Supplementary material for: Ventral and dorsal aspects of the inferior frontal-occipital fasciculus support verbal semantic access and visually-guided behavioural control
Source: Brain Struct Funct. 2023 Dec 9;229(1):207–21. doi: 10.1007/s00429-023-02729-5 (PMC10827863; doi:10.1007/s00429-023-02729-5)
Supplement: Supplementary file 1 — Supplementary file1 (PDF 704 KB) [file 429_2023_2729_MOESM1_ESM.pdf]

SUPPLEMENTARY MATERIALS

Correlation results for task contrasts and left dorsal/ventral IFOF

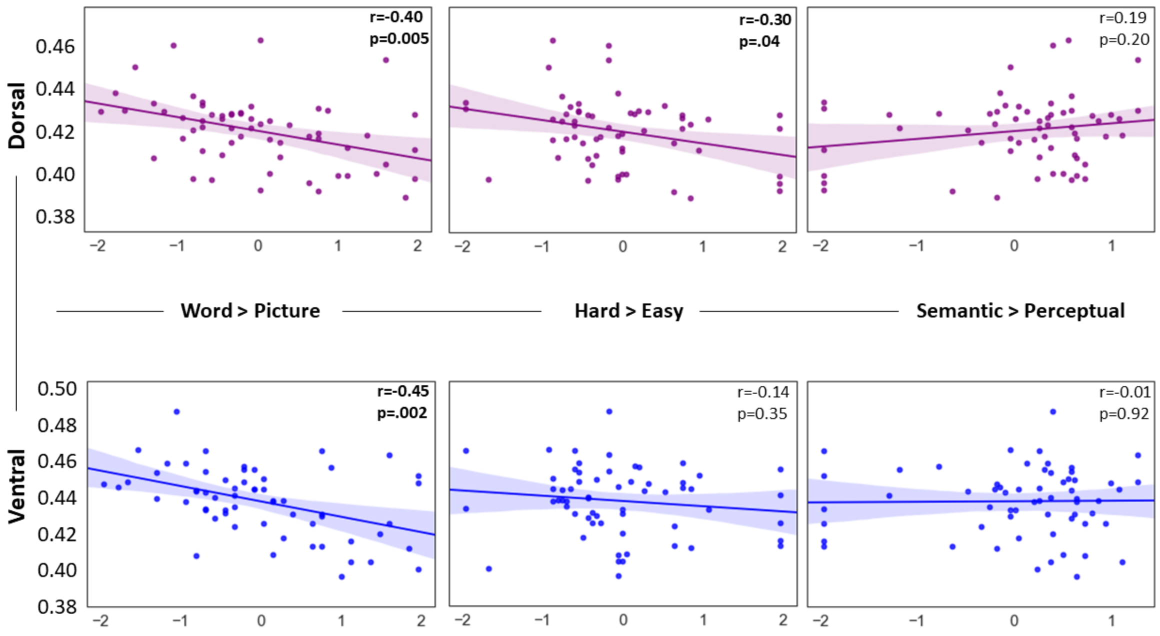

Figure S1. Scatterplots depicting the correlations between Dorsal and Ventral IFOF in LH and the z-scored inverse efficiency scores of the Word > Picture, Hard > Easy and Semantic > Perceptual contrasts.

ANCOVA Results

ID	Type III Sum of Squares	DF	Mean Square	F	Sig.	Effect Size (η_p^2)
			**Analysis 1: Four behavioural covariates by Four IFOF tracts**			
**Task**	0.099	1.674	0.059	2.113	0.135	.037
**Task * IFOF dorsal LEFT**	0.331	1.674	0.198	7.063	0.003	.114
**Task * IFOF ventral LEFT**	0.175	1.674	0.105	3.740	0.035	.064
**Task * IFOF dorsal RIGHT**	0.104	1.674	0.062	2.211	0.124	.039
**Task * IFOF ventral RIGHT**	0.101	1.674	0.060	2.151	0.130	.038
**Error(Task)**	2.578	92.059	0.028			
			**Analysis 2: Four behavioural covariates by Hemispheric Differences in Dorsal and Ventral IFOF tracts**			
**Task**	0.876	1.727	0.508	18.469	0.000	.245
**Task * Dorsal L - R**	0.289	1.727	0.167	6.089	0.005	.097
**Task * Ventral L - R**	0.131	1.727	0.076	2.761	0.076	.046
**Error(Task)**	2.705	98.416	0.027			
			**Analysis 3: Three behavioural contrasts by Four IFOF tracts**			
**Task**	0.095	1.373	0.069	2.437	0.112	.042
**Task * IFOF dorsal LEFT**	0.333	1.373	0.242	8.534	0.002	.134
**Task * IFOF ventral LEFT**	0.180	1.373	0.131	4.630	0.024	.078
**Task * IFOF dorsal RIGHT**	0.101	1.373	0.074	2.589	0.100	.045
**Task * IFOF ventral RIGHT**	0.103	1.373	0.075	2.649	0.096	.046
**Error(Task)**	2.144	75.495	0.028			

Supplementary Table S1. ANCOVAs Results

Pearson Correlation Results

Rank	ID	Original P value	Critical Value	Benjamini-Hochberg Adjusted P value	Significant using an FDR of 0.05?
	**Four behavioural covariates by Left IFOF tracts (Figure 3)**				
**1**	Word Efficiency by Dorsal Left IFOF	0.000261	0.00625	0.002085	Yes
**2**	Word Efficiency by Ventral Left IFOF	0.000743	0.0125	0.002972	Yes
**3**	Hard Efficiency by Dorsal Left IFOF	0.012867	0.01875	0.034311	Yes
**4**	Easy Efficiency by Dorsal Left IFOF	0.063195	0.025	0.126391	No
**5**	Picture Efficiency by Dorsal Left IFOF	0.067295	0.03125	0.107672	No
**6**	Picture Efficiency by Ventral Left IFOF	0.153714	0.0375	0.204952	No
**7**	Easy Efficiency by Ventral Left IFOF	0.385387	0.04375	0.440443	No
**8**	Hard Efficiency by Ventral Left IFOF	0.400981	0.05	0.400981	No
	**Four behavioural covariates by Hemispheric Differences in Dorsal IFOF tracts (Figure 4)**				
**1**	Easy Efficiency by Dorsal left - right IFOF	0.006246	0.0125	0.024983	Yes
**2**	Word Efficiency by Dorsal left - right IFOF	0.006796	0.025	0.013593	Yes
**3**	Hard Efficiency by Dorsal left - right IFOF	0.008606	0.0375	0.011475	Yes
**4**	Picture Efficiency by Dorsal left - right IFOF	0.078505	0.05	0.078505	No
	**Three behavioural contrasts by Left IFOF tracts (Figure S1)**				
**1**	Word > Picture by Ventral Left IFOF	0.000353	0.008333	0.002118	Yes
**2**	Word > Picture by Dorsal Left IFOF	0.001671	0.016667	0.005012	Yes
**3**	Hard > Easy by Dorsal Left IFOF	0.020119	0.025	0.040238	Yes
**4**	Semantic > Perceptual by Dorsal Left IFOF	0.135645	0.033333	0.203467	No
**5**	Hard > Easy by Ventral Left IFOF	0.298884	0.041667	0.35866	No
**6**	Semantic > Perceptual by Ventral Left IFOF	0.920084	0.05	0.920084	No

Supplementary Table S2. Exact p values and FDR-corrected values for the correlations of behavioural efficiency and IFOF tract integrity

Output of the *cocor* analysis for comparing the correlations of the hard perceptual condition with the integrity of left dorsal versus left ventral IFOF

INPUT:

cocor.dep.groups.overlap(r.jk=-0.32, r.jh=-0.11, r.kh=+0.75, n=60, alternative="two.sided", alpha=0.05, conf.level=0.95, null.value=0)

OUTPUT:

Results of a comparison of two overlapping correlations based on dependent groups

Comparison between r.jk = -0.32 and r.jh = -0.11

Difference: r.jk - r.jh = -0.21

Related correlation: r.kh = 0.75

Group size: n = 60

Null hypothesis: r.jk is equal to r.jh

Alternative hypothesis: r.jk is not equal to r.jh (two-sided)

Alpha: 0.05

pearson1898: Pearson and Filon's z (1898)

z = -2.3861, p-value = 0.0170

Null hypothesis rejected

hotelling1940: Hotelling's t (1940)

t = -2.4193, df = 57, p-value = 0.0188

Null hypothesis rejected

williams1959: Williams' t (1959)

t = -2.4181, df = 57, p-value = 0.0188

Null hypothesis rejected

olkin1967: Olkin's z (1967)

z = -2.3861, p-value = 0.0170

Null hypothesis rejected

dunn1969: Dunn and Clark's z (1969)

z = -2.3273, p-value = 0.0199

Null hypothesis rejected

hendrickson1970: Hendrickson, Stanley, and Hills' (1970) modification of Williams' t (1959)

t = -2.4193, df = 57, p-value = 0.0188

Null hypothesis rejected

steiger1980: Steiger's (1980) modification of Dunn and Clark's z (1969) using average correlations

z = -2.3136, p-value = 0.0207

Null hypothesis rejected

meng1992: Meng, Rosenthal, and Rubin's z (1992)

z = -2.3019, p-value = 0.0213

Null hypothesis rejected

95% confidence interval for r.jk - r.jh: -0.4095 -0.0329

Null hypothesis rejected (Interval does not include 0)

hittner2003: Hittner, May, and Silver's (2003) modification of Dunn and Clark's z (1969) using a backtransformed average Fisher's (1921) Z procedure

z = -2.3124, p-value = 0.0208

Null hypothesis rejected

zou2007: Zou's (2007) confidence interval

95% confidence interval for r.jk - r.jh: -0.3839 -0.0334

Null hypothesis rejected (Interval does not include 0)

Accuracy Analysis

Although the focus of our main analysis was inhibition efficiency, indexed through inverse efficiency scores, we also analysed accuracy to confirm the difficulty effects, since difficult trials should be less accurate. In the data from Experiment 2, analysed together with structural connectivity in the present study, the Word trials were more demanding than the Picture trials ($t(59) = -11.6$, $p<.0001$, Cohen's $d = 1.5$), and the Perceptual Hard trials were more demanding than the Perceptual Easy trials ($t(59) = -6.68$, $p<.0001$, Cohen's $d = .86$). In this analysis, behavioural performance was not matched for Word and Perceptual Hard conditions ($t(59) = 2.77$, $p=.028$, Cohen's $d = .36$) nor for the Picture and Perceptual Easy conditions ($t(59) = 3.72$, $p=.002$, Cohen's $d = .48$), with both perceptual conditions being harder than the semantic conditions.

Fractional Anisotropy Analysis

In order to document differences between the IFOF subdivisions both within and across hemispheres, we conducted a series of paired t-tests on Fractional Anisotropy (FA) values examining between-hemisphere differences (contrasting left versus right for dorsal, ventral and total IFOF sub-tracts separately) and within-hemisphere differences (contrasting dorsal versus ventral tracts for each hemisphere separately); we also correlated left and right dorsal and ventral IFOF subtracts to examine their similarities. Between hemispheres, there was a significant difference in the FA values of the dorsal IFOF, with the right hemisphere showing greater values ($t=-2.4$, $p=.02$). There were no hemispheric differences for the ventral or total FA values of the IFOF ($p>.05$). Within hemispheres, there was a significant difference in the FA values of dorsal and ventral IFOF, with ventral IFOF showing greater values in both hemispheres (dorsal > ventral left: $t=-10.3$, right: $t=-8.9$; both $p<.0001$). Within-hemisphere correlations in tract strength were high (Pearson $r = 0.75 – 0.77$), while between hemisphere correlations were low to moderate (Pearson $r = 0.24 – 0.58$; see correlation matrix table below.

	Left dorsal	Left ventral	Right dorsal	Right ventral
Left dorsal	1	0.747101	0.270359	0.239544
Left ventral	0.747101	1	0.420798	0.577182
Right dorsal	0.270359	0.420798	1	0.770807
Right ventral	0.239544	0.577182	0.770807	1

Supplementary Table S3. Correlation matrix of fractional anisotropy values of the IFOF subdivisions

Approach used to segment the Inferior Frontal-Occipital Fasciculus into its dorsal and ventral components applied to an example case

1. We used ExploreDTI for this workflow. In order to isolate the full IFOF tract, we place two AND ROIs encompassing all the white matter fibres in coronal slices. One was placed at the occipital region (the largest one in Fig. S2 below), the second was placed immediately anterior to genu of corpus callosum.
2. We placed a NOT ROI using a sagittal view, separating both hemispheres to exclude crossing fibres (i.e., left to right hemisphere fibres and vice versa). This ROI is shown in red in Fig. S2

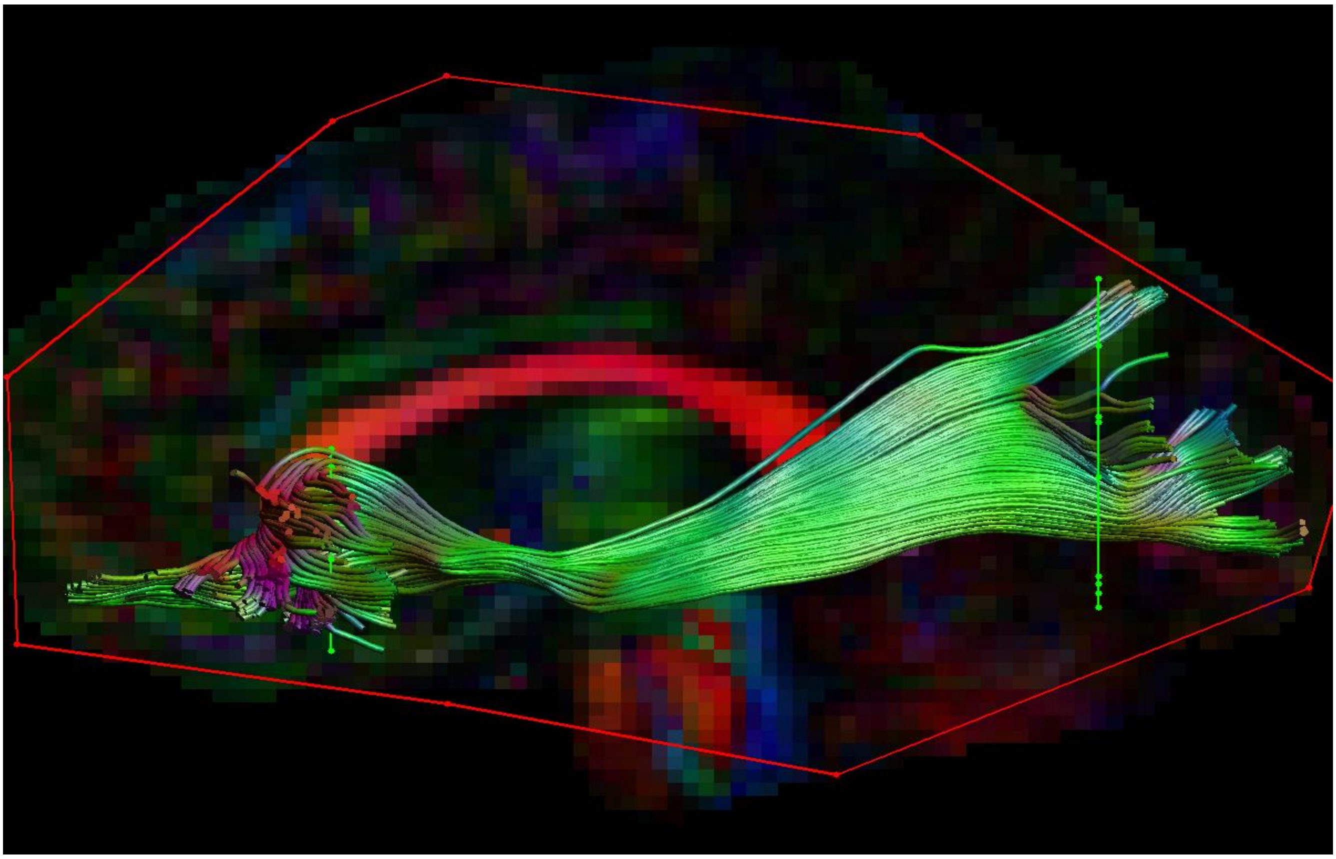

Figure S2. Placement of the two AND ROIs and the NOT ROI used to isolate the full IFOF tract in one participant. The AND ROIs are shown in green whilst the NOT ROI is shown in red.

3. We then compared the resulting tract with the IFOF template from the Catani and Thiebaut de Schotten (2008) Atlas, manually segmented, to assess the quality of the dissection.

4. As illustrated in Figure S3, the clearest division between dorsal and ventral IFOF tracts can usually be found in the occipital lobe. The ventral aspect encompasses fibres that run parallel to the optic radiation, whilst the dorsal runs more parallel to the vertical occipital fasciculus. This is ideal for placing two separate AND ROIs (shown in green in Figure S3, separated by a thick orange line), allowing us to segment the dorsal and ventral components.

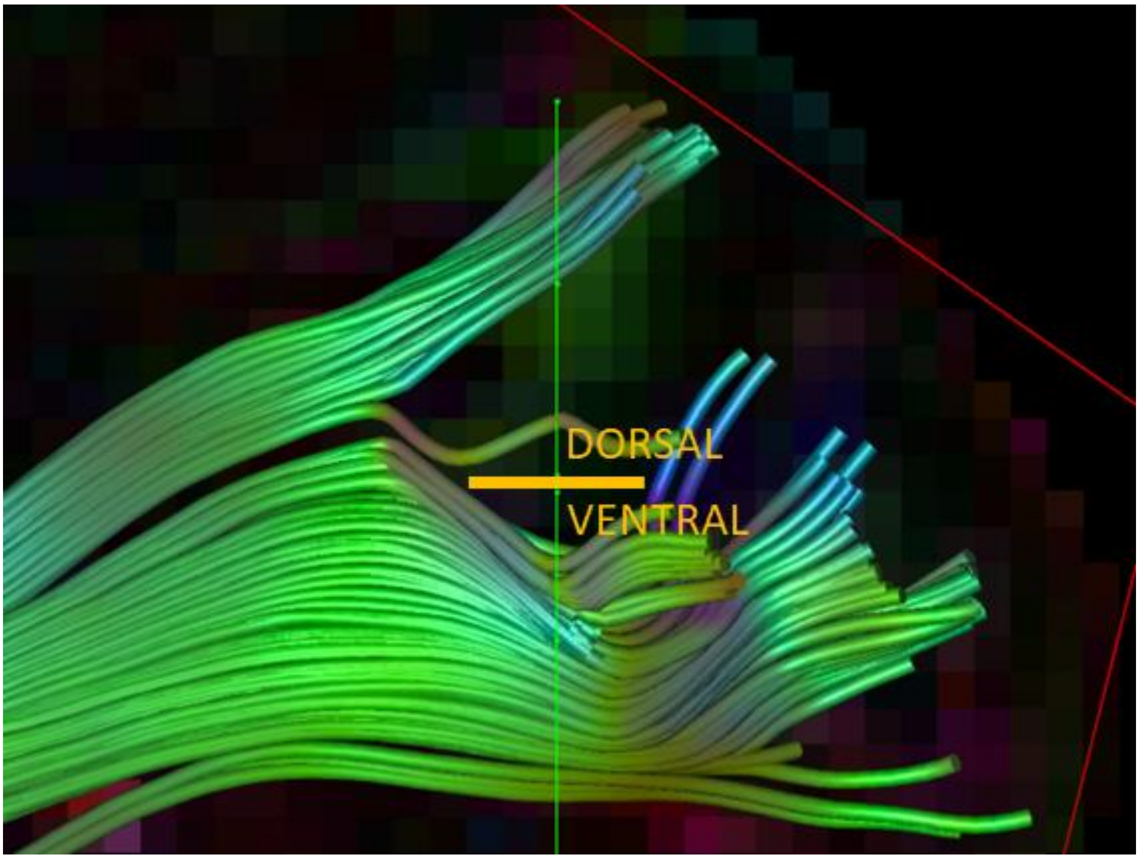

Figure S3. Zoom-in on the occipital end of the full IFOF tract from Figure S2 depicting the separation of the dorsal and ventral IFOF fibres in the occipital lobe, ideal for placing two separate ROIs to isolate the dorsal and ventral IFOF components.

5. In order to be able to place the AND ROIs that capture this division, it is necessary to first have the whole IFOF tract. After this, we can extract the Dorsal IFOF first, by placing the AND ROI labelled 'DORSAL' in Figure S3 in addition to the ROIs shown in Figure S2. The Dorsal IFOF tract resulting from this process can be seen in Figure S4.

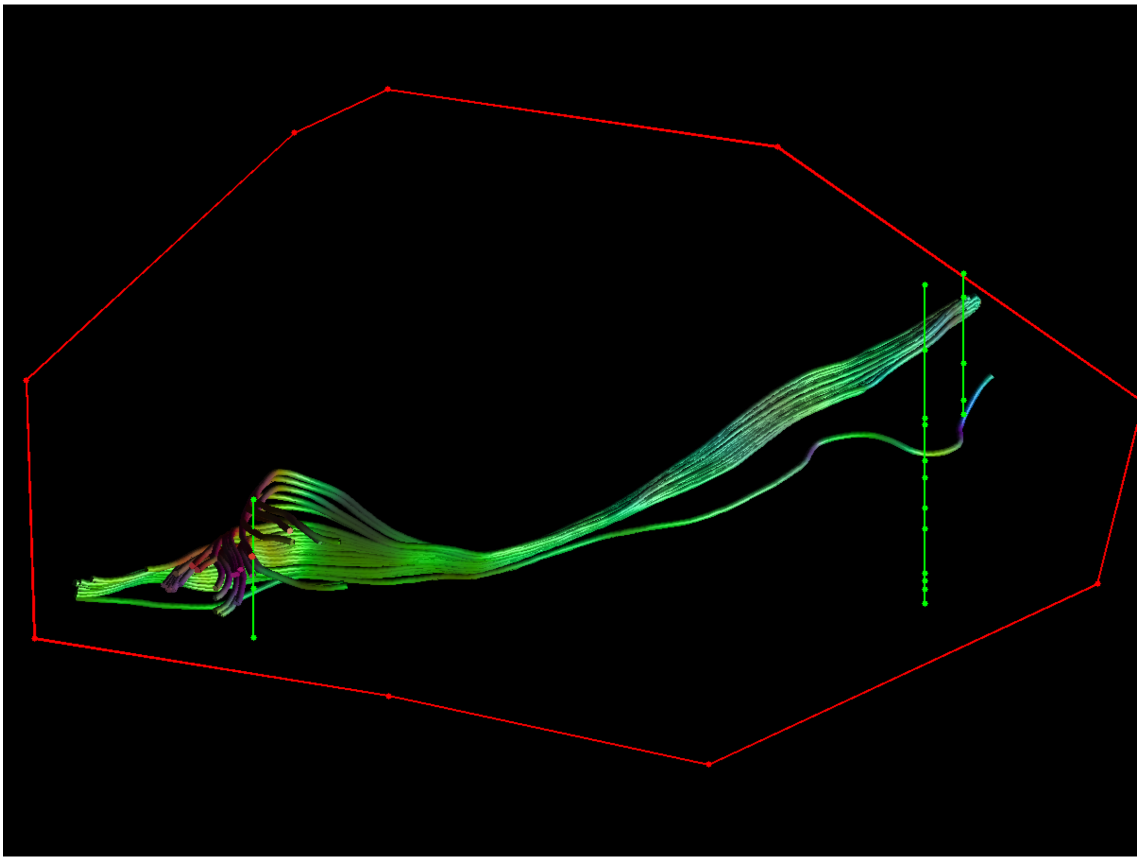

Figure S4. Dorsal IFOF tract extraction in one participant, with all the ROIs used to perform the extraction. The AND ROIs are shown in green, whilst the NOT ROI is shown in red.

6. The Ventral IFOF tract can be extracted in a similar fashion, by placing the AND ROI labelled ‘VENTRAL’ in Figure S3 in addition to the ROIs shown in Figure S2. The Ventral IFOF tract resulting from this process can be seen in Figure S5.

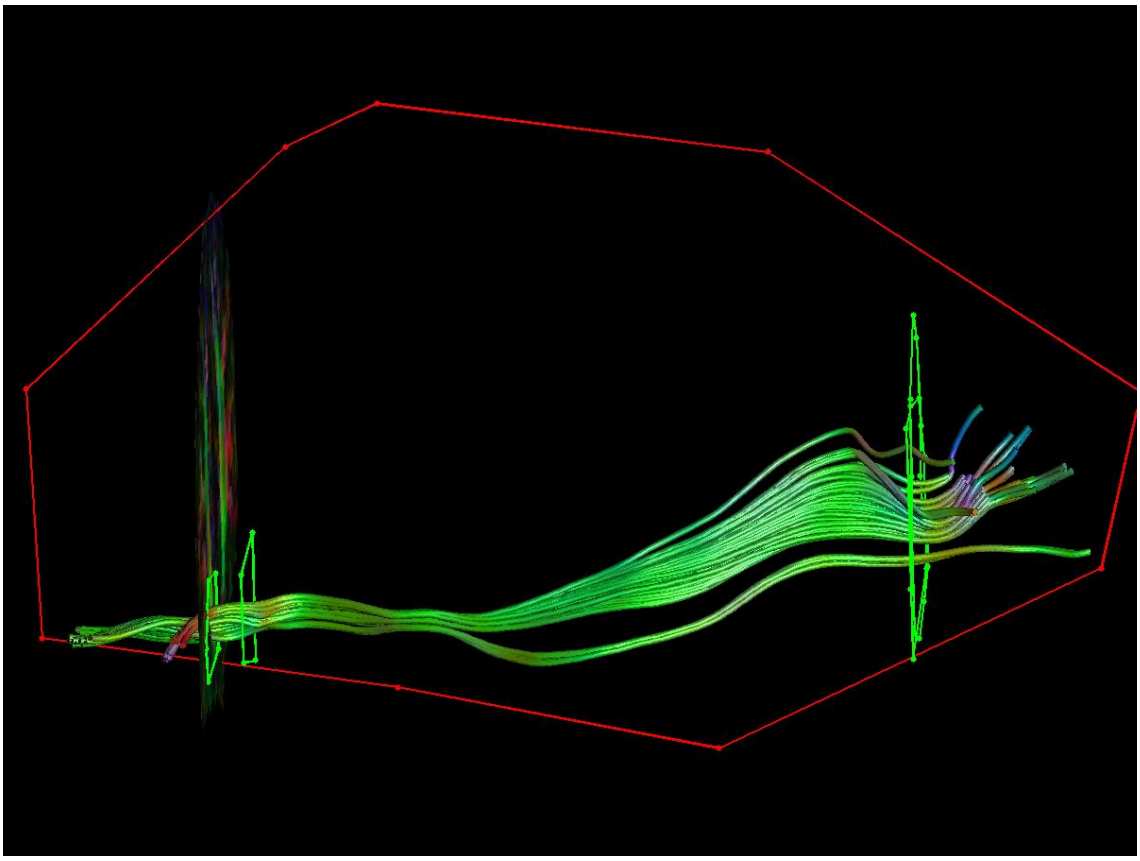

Figure S5. Ventral IFOF tract extraction in one participant, with all the ROIs used to perform the extraction. The AND ROIs are shown in green, whilst the NOT ROI is shown in red.

7. In some cases, further NOT ROIs were necessary to eliminate spurious fibres from adjacent tracts. This was not needed for this particular case, so it is not shown.
8. Utilizing both tracts, ventral and dorsal, we can now rebuild the IFOF using two different colours for visualisation. This method was used to produce Figure 2.
